# Natural immunogenic properties of bioinformatically predicted linear B-cell epitopes of dengue envelope and pre-membrane proteins

**DOI:** 10.1186/s12865-021-00462-4

**Published:** 2021-11-03

**Authors:** Mahesha N. Nadugala, Chandima Jeewandara, Ramesh S. Jadi, Gathsaurie N. Malavige, Aravinda M. de Silva, Prasad H. Premaratne, Charitha L. Goonasekara

**Affiliations:** 1 Faculty of Medicine, General Sir John Kotelawala Defence University, Ratmalana, Sri Lanka; 2 Allergy Immunology and Cell Biology Unit, University of Sri Jayewardanapura, Gangodawila, Sri Lanka; 3 Department of Microbiology and Immunology, University of North Carolina, Chapel Hill, NC 27599 USA; 4 Centre for Dengue Research, University of Sri Jayawardanapura, Gangodawila, Sri Lanka

**Keywords:** Dengue, E protein, prM protein, Natural infections, Microneutralization

## Abstract

**Background:**

The natural antibody responses to B-cell epitopes from dengue structural proteins were assessed using immune sera from people having well-defined past dengue infections with one of the four serotypes.

**Method:**

Based on an immune-computational analysis previously conducted, nineteen epitopes from the envelope (E) and eight epitopes from pre-membrane (prM), which were more than 50% conserved across all the four DENV serotypes, were selected. Peptides to represent these B-cell epitopes were obtained from commercially available arrays, and were subjected to enzyme linked immunosorbent assay with sera obtained from dengue seropositive healthy volunteers (DENV1 n = 12: DENV2 n = 12: DENV3 n = 12 and DENV4 n = 12), and 10 dengue seronegative healthy volunteers from Sri Lanka. The cut-off value for the positive antibody response was set by taking the mean response of a peptide to the negative sera plus three standard deviations. The peptides (N = 7) showing the broad immune responses were used to generate antibodies in three mice (Balb/c) batches. The mice antisera were then subjected to microneutralization assays against all the four DENV serotypes. An EC_50_ viral neutralization ≥ 40 times the serum dilution was considered as neutralizing.

**Results:**

Five of the E-peptide and two prM peptides were recognised by most individuls exposed to infections with each of the four serotypes, showing a serotype cross-reactive broad antibody response. The mice immune sera against the peptides representing the five E protein epitopes neutralized all the four DENV serotypes. Two of these five epitopes are from the Domain II, whereas one of them includes the whole bc-loop region.

**Conclusion:**

The antibody responses of highly conserved epitopes across the serotypes, were broadly responsive with sera of all four DENV serotypes collected from individuals infected with only one DENV serotype. Weakly conserved epitopes showed rather specific antibody responses dominated by one or few serotypes.

**Supplementary Information:**

The online version contains supplementary material available at 10.1186/s12865-021-00462-4.

## Background

Dengue viral (DENV) infections are considered to be one of the rapidly spread mosquito borne viral infections in all regions of the World Health Organisation (WHO) placing half of the people at risk [1, 2]. The Sri Lankan population has been exposed to dengue virus for decades, but severe forms of dengue infections were rare until 1989 [3]. Since then, Sri Lanka has been experiencing yearly epidemics of dengue hemorrhagic fever (DHF), with the number of cases rising each year. More than thirty thousand suspected dengue cases have been reported to the Epidemiology Unit of Ministry of Health from all over the country in 2020 [4].

DENV is a positive-sense RNA virus [5] coding for three structural proteins—capsid (C), pre-membrane (prM) and envelope (E) and seven non-structural proteins [6]. Of these, E and the prM, which are exposed on the surface of the virion, play an important role in virus entry into host cell, and also principally are the targets of host antibodies [7–14].

DENV has four serotypes (DENV1-4) and belongs to family Flaviviridae [15, 16]. Infections with natural DENV produces high titer of neutralizing antibodies, which is an important aspect of protective immune response [17–19]. In heterotypic infections of DENV, cross-reactive antibodies from the previous infection is said to enhance viral infectivity, by forming non-neutralizing complexes with the virus, through a mechanism known as antibody-dependent enhancement [20]. This is considered as a potential complication of dengue vaccine design efforts [21–23]. Mapping of sites on E and prM proteins recognized by natural human antibodies is therefore necessary for the better understanding of dengue immune responses, and thereby for the development of effective  therapeutic options.

In this line, we previously reported the prediction of B-cell epitopes from dengue E and prM proteins, and their conservational analysis using a bioinformatics approach [24]. The present study describes the immunogenic potential of those predicted epitopes that are conserved across and within the four serotypes, during natural dengue infections in the Sri Lankan population. Further, the neutralization potential of the epitopes with broader immunogenic potential across the four serotypes, was measured using mouse model. The B-cell epitopes with broadly immunogenic and neutralizing potential for all four dengue serotypes are discussed with respect to their location on the mature virus.

## Results

**Fig. 1:**
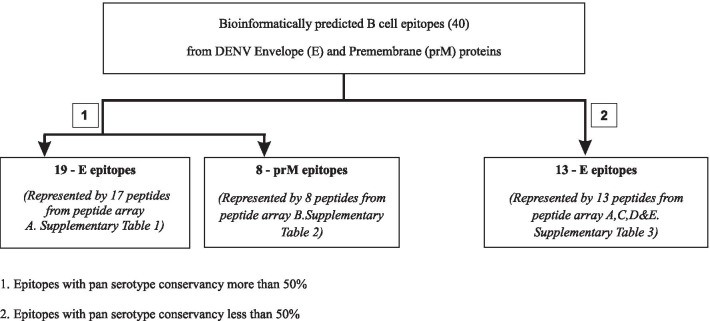
Categorization of predicted epitopes. Array A—E protein peptide array of DENV1 [DENV1 (Singapore/S275/1990 (NR-4551))] (Additional file 1: Table 1). Array B—prM protein peptide array of DENV2 [DENV2 (New Guinea C (NR-506))] (Additional file 2: Table 2). Array C—E protein peptide array of DENV2 (New Guinea C (NR-507)) Array D—E protein peptide array of DENV3 (Sleman/1978 (NR-511)), Array E—E protein peptide array of DENV4 (Dominica/814669/1981 (NR-512))] (Additional file 3: Table 3)

Bioinformatically predicted B-cell epitopes of dengue envelope and pre-membrane proteins were analyzed for their responses to natural antibodies generated during dengue infection in people. In our previous study, we predicted linear B-cell epitopes from DENV E and prM proteins using three different epitope prediction tools [24]. Each epitope was represented by a peptide which was selected from a protein peptide array of a respective DENV isolate of a given serotype (DENV1 for E protein and DENV2 for prM protein) as described under the methodology section. Predicted epitopes were categorized based on the amino acid variability of the epitopes across the four DENV serotypes [24] as given in Fig. 1. These peptides were then subjected to indirect ELISA assays with sera collected from healthy volunteers previously infected with DENV.

### Natural antibody responses to B-cell epitopes with a pan serotype conservancy more than 50%

Antibody responses of the E protein peptides to human sera collected from individuals, who have been naturally infected with only one DENV serotype, as assayed by ELISA, are given in Table 1. A positive antibody response is an ELISA measurement that is above the cut-off value indicates the presence of antibodies against the particular epitope. Five peptides, P4/E, P5/E, P14/E, P15/E and P17/E showed high positive antibody responses to sera of all the four DENV serotypes. The corresponding epitopes therefore appears to be broadly immunogenic, across all the four DENV serotypes. This is also reflected in the fact that these peptides show high positive antibody responses for sera of heterogeneous infections (more than 67%). The peptides P2/E, P3/E, P12/E, P13/E and P16/E showed positive responses with sera of only three of the four DENV serotypes. The peptides P1/E and P11/E, showed some positive responses with sera of either one or two DENV serotypes, while P6/E, P7/E, P8/E, P9/E and P10/E showed no positive responses with sera of any of the DENV serotypes.

**Table 1:** Percentage positive antibody responses of human sera for peptides that represent E protein epitopes with a pan serotype conservancy more than 50%

Peptide ID (location)	Sera infected with one serotype				Sera infected with two serotypes
	DENV1 serotype	DENV2 serotype	DENV3 serotype	DENV4 serotype	
P1/E (7–23)	1 8%	0 0%	0 0%	1 8%	0 0%
P2/E (30–46)	12 100%	9 75%	6 50%	0 0%	4 33%
P3/E (60–76)	10 83%	0 0%	9 75%	12 100%	5 42%
P4/E (72–88)	12 100%	12 100%	12 100%	12 100%	11 91%
P5/E (89–104)	12 100%	12 100%	12 100%	12 100%	10 83%
P6/E (110–126)	0 0%	0 0%	0 0%	0 0%	0 0%
P7/E (178–194)	0 0%	0 0%	0 0%	0 0%	0 0%
P8/E (238–254)	0 0%	0 0%	0 0%	0 0%	0 0%
P9/E (255–271)	0 0%	0 0%	0 0%	0 0%	0 0%
P10/E (279–295)	0 0%	0 0%	0 0%	0 0%	0 0%
P11/E (308–324)	6 50%	0 0%	5 42%	0 0%	0 0%
P12/E (371–387)	9 75%	1 8%	9 75%	0 0%	2 17%
P13/E (377–393)	0 0%	12 100%	9 75%	9 75%	6 50%
P14/E (394–410)	10 83%	10 83%	12 100%	12 100%	12 100%
P15/E (418–434)	12 100%	12 100%	12 100%	12 100%	12 100%
P16/E (424–440)	0 0%	12 100%	10 83%	10 83%	5 42%
P17/E (458–474)	10 83%	9 75%	9 75%	9 75%	8 67%

Number of sera samples giving positive responses out of twelve sera samples tested per serotype (DENV1, DENV2, DENV3, DENV4), and its percentage (%) are shown. The number of total tested sera samples infected with two serotypes are also twelve. The corresponding epitope/peptide sequences from cross serotypes are based on the peptide arrays; DENV1 (Singapore/S275/1990 (NR-4551)

Antibody response pattern from prM peptides is shown in Table 2. Sera of all four serotypes, collected from individuals infected with only one DENV serotype, showed positive antibody responses to only two peptides: P1/prM and P7/prM. Epitopes representing these two peptides, therefore showed a broader immunogenic nature across the four DENV serotypes. However, P1/prM did not show a very high antibody response with the sera of heterogeneous infection (only 42%). There was no antibody response against the: P2/prM, P3/prM and P5/prM peptides. Only the sera of DENV2 showed a positive antibody response to P4/prM peptide while only the sera of DENV4 and DENV3 showed a positive antibody response to P6/prM and P8/prM peptides respectively.

**Table 2:** Percentage positive antibody responses of sera for peptides that represent prM protein epitopes with a pan serotype conservancy > 50%

Peptide ID (location)	Sera infected with one serotype				Sera infected with two serotypes
	DENV1 serotype	DENV2 serotype	DENV3 serotype	DENV4 serotype	
P1/prM (1–16)	12 100%	12 100%	12 100%	12 100%	5 42%
P2/prM (15–31)	0 0%	0 0%	0 0%	0 0%	0 0%
P3/prM (43–57)	0 0%	0 0%	0 0%	0 0%	0 0%
P4/prM (55–74)	0 0%	1 8%	0 0%	0 0%	0 0%
P5/prM (73–90)	0 0%	0 0%	0 0%	0 0%	0 0%
P6/prM (88–107)	0 0%	0 0%	0 0%	12 100%	3 25%
P7/prM (102–119)	12 100%	12 100%	12 100%	12 100%	8 67%
P8/prM (126–142)	0 0%	0 0%	2 17%	0 0%	0 0%

Number of sera samples giving positive responses out of twelve sera samples tested per serotype (DENV1, DENV2, DENV3, DENV4), and its percentage (%) are shown. The number of total tested sera samples infected with two serotypes are also twelve. The corresponding epitope/peptide sequences from cross serotypes are based on the peptide arrays; DENV2 (New Guinea C (NR-506))

### Natural antibody responses of B-cell epitopes with a pan serotype conservancy less than  50%

The four peptide sequences from the four DENV serotypes, representing each epitope, with less than 50% pan serotype conservation, were subjected to ELISA assays with sera collected from individuals who are infected with one of the four serotypes. These epitopes are of high interest as serotype specific diagnostic markers. The antibody responses of these peptides, to human sera of different serotypes, are shown in Table 3. The responses shown by the four peptide sequences of a given epitope varied across the four serotypes. For some epitopes, peptides have shown a positive antibody response, which is more or less only towards the sera of the same type, showing a serotype specific response. Some peptides have shown positives responses to sera of other serotypes as well, indicating a serotype cross-reactive antibody response. A peptide sequence showing positive antibody responses with sera of the same serotype and negative responses with sera of other serotypes is considered as a serotype specific peptide /epitope. The specificities and sensitivities of the antibody responses shown by the four peptides of a given epitope towards sera of its own type are shown in Table 3.

**Table 3:** The antibody responses of the four representative DENV peptides of E protein epitopes with less than 50% pan serotype conservancy

Epitope ID/peptide ID		Serotype of sera				% Sensitivity	% Specificity
		DENV1	DENV2	DENV3	DENV4		
EP4/E	DENV1	0 0%	0 0%	0 0%	0 0%	0	100
	DENV2	0 0%	0 0%	0 0%	0 0%	0	100
	DENV3	1 8%	0 0%	2 17%	0 0%	17	97.2
	DENV4	0 0%	1 8%	0 0%	4 33%	33	97.2
EP8/E	DENV1	10 83%	2 17%	5 42%	1 8%	83	77.8
	DENV2	5 42%	4 33%	0 0%	2 17%	33	80
	DENV3	8 67%	1 8%	8 67%	0 0%	67	75
	DENV4	0 0%	0 0%	0 0%	0 0%	0	100
EP9/E	DENV1	0 0%	0 0%	0 0%	0 0%	0	100
	DENV2	0 0%	0 0%	0 0%	0 0%	0	100
	DENV3	0 0%	0 0%	0 0%	0 0%	0	100
	DENV4	0 0%	0 0%	0 0%	0 0%	0	100
EP11-12/E	DENV1	10 83%	0 0%	1 8%	0 0%	83	97.2
	DENV2	0 0%	10 83%	0 0%	0 0%	83	100
	DENV3	0 0%	2 17%	5 42%	3 25%	42	86
	DENV4	0 0%	0 0%	4 33%	4 33%	33	89
EP14/E	DENV1	2 17%	0 0%	0 0%	1 8%	17	97.2
	DENV2	2 17%	12 100%	12 100%	0 0%	100	61
	DENV3	0 0%	10 83%	11 92%	0 0%	92	72
	DENV4	0 0%	0 0%	0 0%	11 92%	92	100
EP17/E	DENV1	3 25%	1 8%	4 33%	1 8%	25	83
	DENV2	0 0%	6 50%	0 0%	5 42%	50	86
	DENV3	0 0%	1 8%	10 83%	4 33%	83	86
	DENV4	0 0%	1 8%	10 83%	4 33%	33	30
EP20/E	DENV1	12 100%	0 0%	0 0%	0 0%	100	100
	DENV2	0 0%	8 67%	0 0%	1 8%	67	97.2
	DENV3	2 17%	0 0%	11 92%	0 0%	92	94.4
	DENV4	0 0%	0 0%	0 0%	10 83%	83	100
EP21/E	DENV1	2 17%	0 0%	0 0%	0 0%	17	100
	DENV2	0 0%	0 0%	0 0%	0 0%	0	100
	DENV3	0 0%	0 0%	0 0%	0 0%	0	100
	DENV4	0 0%	0 0%	0 0%	0 0%	0	100
EP22-23/E	DENV1	4 33%	0 0%	3 25%	0 0%	33	91.6
	DENV2	0 0%	6 50%	4 33%	1 8%	50	86
	DENV3	1 8%	0 0%	10 83%	2 17%	83	91.6
	DENV4	0 0%	1 8%	1 8%	8 67%	67	94.4
EP25/E	DENV1	10 83%	5 42%	12 100%	1 8%	83	50
	DENV2	0 0%	7 58%	1 8%	8 67%	58	75
	DENV3	2 17%	9 75%	12 100%	2 17%	100	63.8
	DENV4	9 75%	4 33%	3 25%	12 100%	100	55.5
EP30/E (first half of the epitope)	DENV1	5 42%	2 17%	7 58%	1 8%	47	72.2
	DENV2	12 100%	12 100%	12 100%	12 100%	100	0
	DENV3	12 100%	12 100%	12 100%	12 100%	100	0
	DENV4	12 100%	11 92%	10 83%	12 100%	100	8.3
EP30/E (latter half of the epitope)	DENV1	0 0%	0 0%	0 0%	0 0%	0	100
	DENV2	0 0%	0 0%	0 0%	0 0%	0	100
	DENV3	0 0%	0 0%	0 0%	0 0%	0	100
	DENV4	0 0%	0 0%	0 0%	0 0%	0	100

Number of sera samples giving positive responses out of 12 sera samples tested per serotype (DENV1, DENV2, DENV3, DENV4), and its percentage (%) are shown. The corresponding epitope/peptide sequences from cross serotypes are based on the peptide arrays; DENV1 (Singapore/S275/1990 (NR-4551), DENV2 (New Guinea C (NR-507), DENV3 (Sleman/1978 (NR-511)), DENV4 (Dominica/814669/1981 (NR-512)

In this regard, the four peptides representing the epitope, EP20/E, is fairly serotype specific. Antibody responses of its peptide sequences from DENV1, DENV2 and DENV4 showed highly specific responses towards sera of the respective serotype. Therefore, the DENV1, -3, and -4 peptides of EP20/E have very high sensitivities and specificities, towards being responsive only to sera of DENV1, -2 or -4 respectively, but not to sera of other types. The sensitivity of the responses by DENV2 peptide of EP20/E was moderate. As such, there were other peptide sequences which have shown serotype specific responses showing fairly high sensitivities and specificities. For example, DENV1 and DENV2 peptides of EP11-12/E, DENV4 peptide of EP14/E, and DENV3 peptide of EP22-23/E, showed serotype specific antibody responses. These peptide sequences have the potential to be developed as serotype specific diagnostic markers for specific identification of the serotype of infection of a dengue infected sera.

### Antibody responses to peptide sequences of all four DENV serotypes of some B-cell epitopes from E protein

Of the nineteen  E protein epitopes with more than 50% pan serotype conservation, nine of the representative peptides (selected from a E protein peptide array from DENV1) showed positive antibody responses to sera of four serotypes or three serotypes. These were subjected to ELISA assays using the representative peptides sequences from other three DENV serotypes as well. This experiment was expected to verify the high conservation of these epitopes across the four DENV serotypes, and their broader immunogenicity against minor variations in the amino acid sequences across serotypes/strains. Epitopes that are positively responsive to naturally generated antibodies against all four DENV serotypes can be potential targets for universal vaccine candidates for dengue. The results are given in Table 4.

**Table 4:** Percentage positive antibody responses of the four representative DENV peptides of broadly immunogenic E protein epitopes to human sera of four DENV serotypes

Serotype of the sera	P4/E (72–88)	P5/E (89–104)	P14/E (394–410)	P15/E (418–434)	P17/E (458–474)
	DENV1 SRCPTQGEATLVEEQDA	DENV1 NFVCRRTFVDRGWGNG	DENV1 KGSSIGKMFEATARGAR	DENV1 TWADFGSIGGVFTSVGK	DENV1 IGILLTWLGLNSRSTSL
DENV1	12 (100%)	12 (100%)	10 (83%)	12 (100%)	10 (83%)
DENV2	12 (100%)	12 (100%)	10 (83%)	12 (100%)	9 (75%)
DENV3	12 (100%)	12 (100%)	12 (100%)	12 (100%)	9 (75%)
DENV4	12 (100%)	12 (100%)	12 (100%)	12 (100%)	9 (75%)
	DENV2 RCPTQGEPSLNEEQDKRF	DENV2 KRFVCKHSMVDRGWGNGCGL	DENV2 KKGSSIGQMIETTMRGAK	DENV2 AILGDTAWDFGSLGGVF	DENV2 IITWIGMNSRSTSLSVSL
DENV1	12 (100%)	12 (100%)	10 (83%)	0 (0%)	12 (100%)
DENV2	12 (100%)	12 (100%)	10 (83%)	0 (0%)	12 (100%)
DENV3	12 (100%)	12 (100%)	10 (83%)	0 (0%)	12 (100%)
DENV4	12 (100%)	12 (100%)	10 (83%)	0 (0%)	12 (100%)
	DENV3 SRCPTQGEAILPEEQDQNH	DENV3 CKHTYVDRGWGNGCGLF	DENV3 KKGSSIGKMFEATARGAR	DENV3 WDFGSVGGVLNSLGKMVH	DENV3 IGIGVLLTWIGLNSK
DENV1	12 (100%)	0 (0%)	12 (100%)	6 (50%)	0 (0%)
DENV2	12 (100%)	0 (0%)	12 (100%)	9 (75%)	0 (0%)
DENV3	12 (100%)	0 (0%)	12 (100%)	10 (83%)	0 (0%)
DENV4	12 (100%)	0 (0%)	12 (100%)	6 (50%)	3(25%)
	DENV4 TRCPTQGEPYLKEEQDQQY	DENV4 CRRDVVDRGWGNGCGLF	DENV4 SIGKMFESTYRGAKRMAI	DENV4 WDFGSVGGLFTSLGKAVH	DENV4 LVLWIGTNSRNTSMAM
DENV1	2 (17%)	9 (75%)	0 (0%)	10 (83%)	0 (0%)
DENV2	10 (83%)	9 (75%)	0 (0%)	12 (100%)	0 (0%)
DENV3	2 (17%)	10 (83%)	0 (0%)	9 (75%)	1 (8%)
DENV4	12 (100%)	6 (50%)	0 (0%)	9 (75%)	0 (0%)

Serotype of the sera	P2/E (30–46)	P3/E (60–76)	P13/E (377–393)	P16/E (424–440)
	DENV1 CVTTMAKDKPTLDIELL	DENV1 CIEAKISNTTTDSRCPT	DENV1 YIVVGAGEKALKQCWFK	DENV1 SIGGVFTSVGKLVHQVF
DENV1	12 (100%)	10 (83%)	0 (0%)	0 (0%)
DENV2	9 (75%)	0 (0%)	12 (100%)	12 (100%)
DENV3	6 (50%)	9 (100%)	9 (75%)	10 (83%)
DENV4	0 (0%)	12 (100%)	9 (75%)	10 (83%)
	DENV2 SCVTTMAKNKPTLDFELI	DENV2 EIKITPQSSITEAFLTGY	DENV2 FGDSYIIIGVEPGQLKL	DENV2 WDFGSLGGVFTSIGKALH
DENV1	3 (25%)	10 (83%)	1 (8%)	0(0%)
DENV2	12 (100%)	12 (100%)	6 (50%)	0 (0%)
DENV3	6 (50%)	12 (100%)	0 (0%)	0 (0%)
DENV4	0 (0%)	9 (75%)	6 (50%)	0 (0%)
	DENV3 GCVTTMAKNKPTLDIEL	DENV3 LCIEGKITNVTTDSR7	DENV3 KALKINWYKKGSSIGKMF	DENV3 VLNSLGKMVHQIFGSAY
DENV1	0 (0%)	0 (0%)	1 (8%)	0 (0%)
DENV2	0 (0%)	0 (0%)	5 (42%)	2 (17%)
DENV3	4 (33%)	0 (0%)	12 (100%)	0 (0%)
DENV4	5 (42%)	0 (0%)	0 (0%)	3 (25%)
	DENV4 GCVTTMAQGKPTLDFEL	DENV4 SISNITTATRCPTQQGEPY	DENV4 FDSYIVIGVGNSALTLH	DENV4 WDFGSVGGLFTSLGKAVH
DENV1	2 (17%)	9 (75%)	0 (0%)	0 (0%)
DENV2	10 (83%)	3 (25%)	1 (8%)	0 (0%)
DENV3	3 (25%)	10 (83%)	0 (0%)	0 (0%)
DENV4	6 (50%)	12 (100%)	0 (0%)	0 (0%)

Number of sera samples giving positive responses out of twelve sera samples tested per serotype (DENV1, DENV2, DENV3, DENV4), and its percentage (%) are shown. The peptide sequences from each serotype for a respective epitope are shown. The corresponding epitope/peptide sequences from cross serotypes are based on the peptide arrays; DENV1 (Singapore/S275/1990 (NR-4551)), DENV2 (New Guinea C (NR-507)), DENV3 (Sleman/1978 (NR-511)), DENV4 (Dominica/814669/1981 (NR-512))

As the DENV1 peptide sequences of, P4/E, P5/E, P14/E, P15/E and P17/E, their respective peptide sequences from most of the other three DENV serotypes, also showed positive antibody responses to sera across all the four serotypes. In fact, for some peptide sequences, the antibody response to sera of each of the four serotypes were 100%. These peptide sequences are the ones from DENV1, -2 and -3 of P4/E, DENV1 and -2 of P5/E, DENV3 of P14/E, DENV1 of P15/E, and DENV2 of P17/E. This confirms the broader nature of the immunogenicity of these epitopes, irrespective of the slight variations in the peptide sequences from the four serotypes, and identifies the best representative peptide sequence to confer the broadest immunogenicity for a given epitope. In the case of peptides, P2/E, P3/E, P13/E and P16/E, DENV1 peptide sequence of which showed positive antibody responses with sera of only three serotypes, none of the peptide sequences showed 100% antibody responses to sera of all the four serotypes. A reasonably high level of positive responses was observed only for DENV2 sequence counterpart of P3/E.

### Viral neutralizing potential of the broadly immunogenic epitopes

The viral neutralization ability of the antibodies produced against the predicted B-cell epitopes were assessed using viral microneutralization assays. Based on the results of ELISA experiments shown above, out of all 40 predicted B-cell epitopes, five epitopes represented by the peptides, P4/E, P5/E, P14/E, P15/E and P17/E, showed a broader immune response across the sera of four DENV serotypes at 100% sensitivity. Therefore, only these five epitopes were selected for assessing the viral neutralization potential. The E peptide sequence (according to Table 4) of the following DENV serotypes were selected to represent each epitope: DENV1 for P4/E, DENV1 for P5/E, DENV3 for P14/E, DENV1 for P15/E and DENV2 for P17/E.

Mice were immunized using the selected peptides representing each epitope and the collected mice immune sera were subjected to neutralization assay. The viral neutralizing titre of immune sera, obtained from each mouse against the peptides or the controls, are shown in Table 5. A 50% viral neutralization at more than 40 times serum dilution are considered as a titre with the viral neutralization potential. None of the mice immunized with the negative control (adjuvant alone) showed a neutralization titre above 40, indicating no virus neutralization. Whereas all the mice immunized with the positive control (the whole E protein peptides) has shown neutralization titres above 40 against each of the four viruses, showing the potential of virus neutralization by the antibodies generated against whole E protein peptides in mice sera. Mice antisera of the five peptides from the E protein, P4/E, P5/E, P14/E, P15/E and P17/E, also showed neutralization titres above 40 with most of the mice, against each of the four DENV viruses, indicating significant viral neutralization potential against all the four DENV serotypes by the antibodies generated against individual E protein peptides in mice sera.

**Table 5:** The viral neutralization titres of different mice immune sera against different serotypes of DENV

Peptide ID (location)	DENV 1			DENV 2			DENV 3			DENV 4		
	Batch 1	Batch 2	Batch 3	Batch 1	Batch 2	Batch 3	Batch 1	Batch 2	Batch 3	Batch 1	Batch 2	Batch 3
POS	203	471	236	490	324	245	236	1027	369	65	249	50
NEG	20	20	20	20	20	20	20	20	20	20	20	20
P4/E (72–88)	296	321	125	755	797	1250	379	253	218	116	332	185
P5/E (89–104)	139	171	144	829	506	302	267	276	248	81	164	103
P14/E (394–410)	81	346	290	286	541	60	56	86	-	100	137	119
P15/E (418–434)	58	441	749	70	406	344	118	61	968	65	20	794
P17/E (458–574)	257	212	757	239	486	266	361	145	2453	106	182	366

The virus neutralization titres of different mice immune sera against of the four DENV serotypes are shown in the table. The virus neutralization titre is the serum dilution at which 50% virus neutralization (EC_50_). The percentage of virus neutralization is calculated by taking the reduction in the virus titre in the presence of a given immunesera as compared to the virus titre in the absence of immunesera

POS, positive control (the entire DENV E protein). NEG, negative control (PBS and adjuvant); Batch 1–3, different batches of mice

**Fig. 2:**
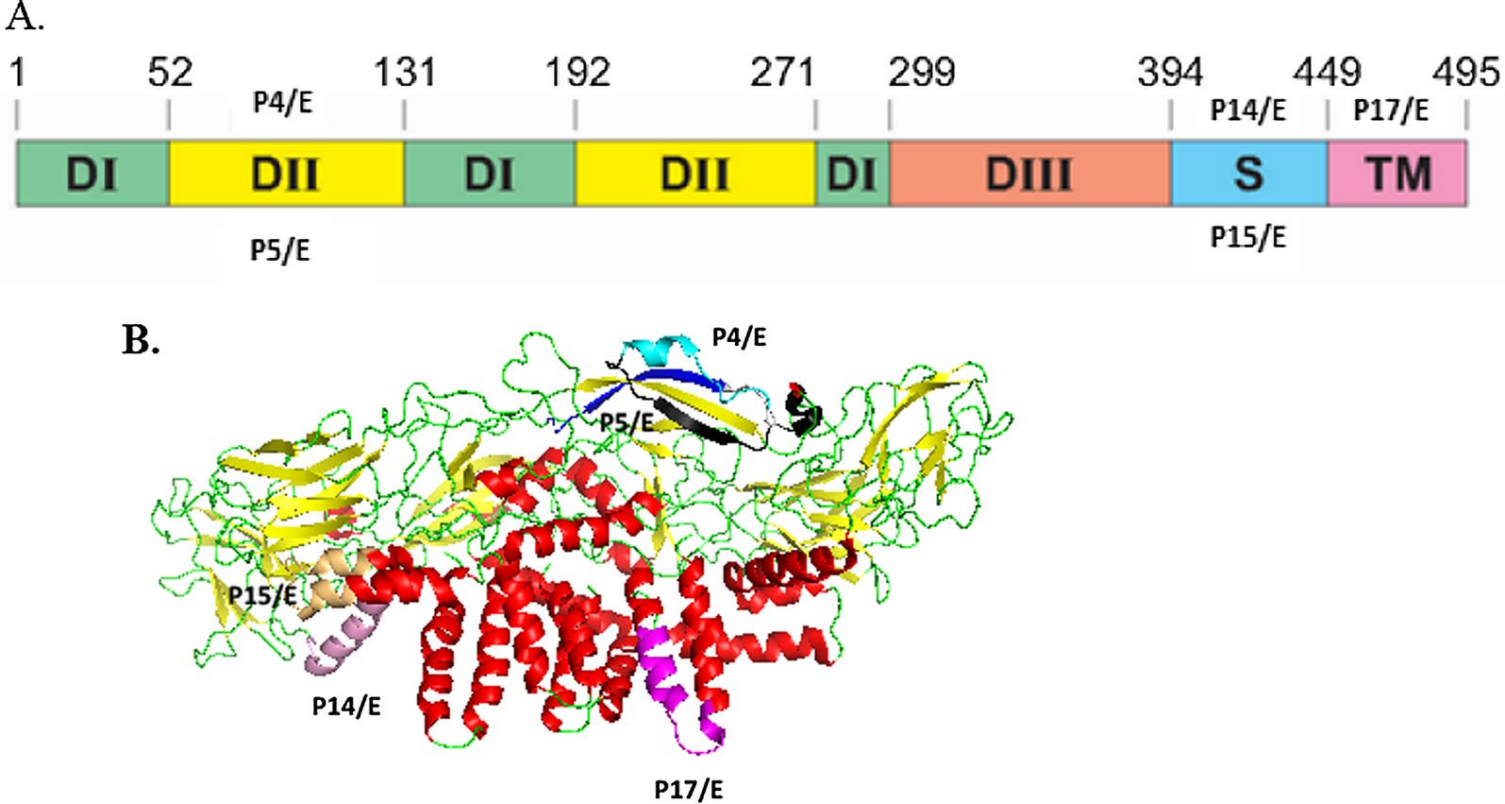
The locations of the broadly immunogenic neutralizing E protein epitopes on the linear structure (**A**), secondary ribbon structure (**B**) of DENV E protein (PDB 3J2P). (P4/E: blue, P5/E: black P14/E: pink, P15/E: brown and P17/E: magenta)

These five E protein epitopes, with broadly immunogenic and viral neutralizing potential were mapped to the solution structure of the E protein. Figure 2 depicts their locations on linear and secondary ribbon structures of the E protein (PDB 3J2P, RCSB Protein Data Bank). Out of these, two are located on the DII of the E protein which include functional important sites in the DENV envelope. The peptide P4/E (72–98) includes the bc loop region (73–79 a.a) and P5/E (89–104) includes fusion loop (FL) region (89–104 a.a), which is also the most conserved region of the DII domain of the E protein. P14/E and P15/E are located on the membrane proximal stem of the E protein and the fifth peptides P17/E is located on the transmembrane anchor.

## Discussion

This study evaluated the immune responses of bioinformatically predicted B-cell epitopes from E and prM proteins to naturally generated antibodies in people infected with dengue virus. In a previous study, we predicted linear B-cell epitopes of DENV E and prM proteins. Linear epitopes however, may not be accessible to antibodies depending on their actual location on the mature virion. Therefore, the actual immunogenicity of these epitopes should be evaluated against antibodies that have been naturally generated against the virus during infections. Our results show strong agreement between E and prM epitopes predicted by different bioinformatic approaches to be antigenic and being recognized by antibodies in people infected with DENV. Most of the epitopes (out of the total 40 predicted epitopes) positively responded to a high percentage of sera collected from individuals infected with DENV, indicating that these epitopes are accessible to antibodies. Some epitopes (P6/E, P7/E, P8/E, P9/E, P10/E and P2/prM, P3/prM, P5/prM) that were evaluated, out of the total 40 predicted epitopes, did not show any antibody responses, probably owing to these epitopes in a buried location in the mature virion.

Further, the levels of conservation in the protein sequences of these epitopes, across the serotypes correlated with the conservation/variability in the antigenic responses. The immunogenic epitopes, which are having high pan serotype conservation, also showed a broader antibody responses across the four DENV serotypes. Some responses were dominated by sera of one or few serotypes in accordance with the low pan serotype conservation levels of those epitopes.

A major strength of our study is the finding of the epitopes with a low pan serotype conservation and having antibody responses towards sera of only one DENV serotype. Such epitopes could be potential candidates as serotype specific diagnostic markers, which can be used to identify the serotype of a DENV infection. For example, the four DENV peptide sequences of EP20/E specifically responded to sera of the same serotype but not with the sera of other serotypes. Of these, the sensitivity towards the sera of the same DENV serotype was also very high with DENV1, DENV3 and DENV4 peptides. These peptide sequences can be used to develop ELISA based diagnostic markers in the specific identification of an infection with the four DENV serotypes. Accordingly, DENV1 peptides of EP 11-12/E & EP20/E, DENV2 peptide of EP 11-12/E, DENV3 peptides of EP22-23/E & P20/E, and DENV4 peptide of EP14/E & EP20/E, can be used in ELISA based diagnostic assay to specifically identify whether the serotype of a DENV infection is DENV1, DENV2, DENV3 or DENV4.

In contrast, certain peptide sequences of five of the B-cell epitopes, with more than 50% pan serotype conservation, showed positive antibody responses with all the sera tested. These sera included all DENV serotypes and each serum is being obtained from an individual infected with only one DENV serotype. The result is quite promising as these peptides are broadly immunogenic across all the four dengue serotypes and having sensitivity of 100% towards DENV. Such an epitope could be a potential target as a marker for dengue group detection. The usability of these peptides, as dengue group or serotype detection markers, is a question to be evaluated further, with more ELISA assays using sera derived from other flaviviral infections.

Another interesting finding of our study is that the peptides represented by the five broadly immunogenic epitopes generated antibodies in mice, which were able to neutralize all the four DENV serotypes. Conserved epitopes with broad immunogenic properties and viral neutralization ability can also be potential targets in epitope-based universal vaccine design against dengue disease. In this line, it is worth exploring the locations of the broadly immunogenic neutralizing epitopes on the native protein. The epitopes represented by P4/E (72–88 aa) and P5/E (89–104 aa) overlap with the highly conserved fusion loop (FL) (97–111 aa) and the bc loop (73–79 aa) of the DII domain of E protein. Fusion loop is involved in fusing with the host cell endosomal membrane, allowing the nucleocapsid of the virion to enter the host cell. Several studies have suggested [25–28], DII and its FL region contains many cross-reactive epitopes eliciting weak to moderate neutralizing monoclonal antibodies. The most significant fusion loop amino acid residues that bind to neutralizing human monoclonal antibodies (hMAB) are W101, L-107 and/or G109 [27]. One of these residues is within the P5/E peptide. Antibodies against the fusion loop as well as the bc loop regions, however, are also said to frequently exhibit neutralizing activities [27, 28], which are disease enhancing rather than protective. The findings of the present study add to this knowledge demonstrating a moderately neutralizing activity for the parts of the fusion/bc loop region on domain II, as represented by the peptides P4/E and P5/E. The viral neutralizing potential of these peptides should be further evaluated by conducting ADE studies of the peptides. The other three broadly immunogenic neutralizing epitopes, represented by P14/E (393–410 aa), P15/E (418–434 aa), and P17/E (458–474 aa), are located on the two α helices in the E protein stem region (396–452 aa), and in the transmembrane domain (452–495 aa), of the E protein [27]. Despite the buried location of these regions in mature virion, our studies clearly demonstrate the presence of antibodies generated against these regions during DENV infections. These peptides, therefore, need to be studied further in detail, to explore their potential and the extent at eliciting immune responses.

## Conclusions

In this study, we demonstrate the natural immunogenic potential of a set of bioinformatically predicted linear B-cell epitopes we previously described. The antibody responses of highly conserved epitopes across the serotypes, were also broadly responsive with sera of all four DENV serotypes collected from individuals infected with only one DENV serotype. Weakly conserved epitopes showed rather specific antibody responses dominated by one or few serotypes. Few such peptides having antibody responses specific towards only one of the four serotypes were identified. Five epitopes on the E protein, showed 100% sensitivity towards sera of all four DENV serotypes as well as viral neutralizing activity. Our results offer new insights into the significance of bioinformatically predicted epitopes from dengue structural proteins in dengue diagnosis and therapeutics.

## Methods

### Selection of Bioinformatically predicted B-Cell Epitopes and their representative peptides

Bioinformatically predicted [24], thirty-two B-cell epitopes representing the dengue E protein and eight representing the prM protein, were selected to investigate their immunogenicity during natural infections, using an in-house ELISA. Peptides that best represent those predicted B-cell epitopes were selected from the peptide arrays obtained from the Biodefense and Emerging Infections Research Repository, National Institutes of Health (NIH). Following arrays were used; E protein peptides [DENV1 (Singapore/S275/1990 (NR-4551)), DENV2 (New Guinea C (NR-507)), DENV3 (Sleman/1978 (NR-511)), DENV4 (Dominica/814669/1981 (NR-512))] and prM protein peptides [DENV2 (New Guinea C (NR-506)]. For all the ELISA experiments, peptides selected from the above peptide arrays were used. For immunization of mice, peptides were commercially  synthesized at 90% purity.

### Collection of human sera

DENV immune sera were obtained from sixty seropositive healthy volunteers residing in Sri Lanka who had experienced natural DENV infections [29]. Infection with dengue was confirmed by NS1, IgM/ IgG ELISA. The serotype of the previous dengue infections of these subjects have been determined as previously described by using cultured T-cell ELISpot assays [29], using a panel of peptides which were found to be highly conserved within a serotype and unique to that serotype, therefore, specifically detects an infection by a given dengue virus serotype [30]. Accordingly, individuals whose T-cells responded to peptides that were unique to a particular serotype were considered as being infected with only that particular serotype. Those who responded to two serotypes were considered as being previously infected with those two serotypes. Out of the sixty samples selected for the study, 48 were from healthy volunteers who had only responded to peptides of one dengue viral serotype and therefore considered to have been previously exposed to infections with only one of the 4 DENV serotypes (n = 12 per serotype). The remaining 12 samples were from healthy volunteers who responded to peptides of two dengue viral serotypes and were considered to have been previously infected with two DENV serotypes. In addition, sera from twelve dengue naive volunteers were also obtained to use as controls. Therefore, each study group is comprised of 12 sera samples with known identity of the type of dengue infection. Inclusion criteria for selecting volunteers are as follows; healthy people between 10 ± 70 years of age, carrying antibodies against one or two serotypes of DENV. Debilitated/bed ridden peoples and peoples below 10 and over 70 years of age were excluded in this study. Pooled sera consisting of ten samples representing all four serotypes were used to prepare the positive control serum sample and ten naive samples were used to prepare negative control sample.

### Indirect ELISA assay

For ELISA [31], 96-well polystyrene plates (Sigma, USA) were used. Peptides dissolved in PBS were added at 1.0 μg/well/100 μl in a carbonate ± biocarbonate buffer (pH 9.6) in duplicate and incubate at 4 °C for 12 h. After the peptide solution was flicked off from wells, the plate was blocked with 200 μl of blocking buffer (10% skimmed milk powder in PBS, pH 7.4, with 0.05% Tween-PBST) at 37 °C for 2 h. Subsequently the plate was washed three times with PBST. Serum samples were diluted 1/100 in blocking buffer, and 100 μl of diluted serum was added to each well. The plate was incubated at 37 °C for 1 h and washed three times with PBST. Anti-Human IgG (whole molecule)-Peroxidase antibody produced in rabbit (Sigma, USA) was diluted 1/1000 blocking solution and added to wells. Then the wells were incubated at 37 °C for 1 h, followed by three washes with PBST. 100 μl of the substrate solution was then added (10 ml of 0.1 citric acid buffer (pH 5.0), 250 μl of ABST stock solution (100 mg of ABTS in 4.5 ml deionised water) and 50 μl of H_2_O_2_. Samples were then incubated at room temperature for 20 min. The plates were read with a microplate reader with a 450 nm filter. The final absorbance result values for each serum sample correspond to the arithmetic mean of the duplicate measurements. The cut-off value for positive immune response was defined using following formula: Cut-off = Mean OD of negative samples + 3 standard deviation (S.D).

### Statistical analyses

The mean OD values obtained for each combination of serotype and the peptide were subjected to General Linear Models (GLM) and Least square (LS) mean separation procedure in SAS 9.1 (SAS Institute, Cary, NC, USA).

### Mice immunization and collection of mice immune sera

Mice were immunized with the peptides, to generate antisera, to test for the viral neutralizing potential of the B-cell epitopes. Five E protein  peptides, which showed broad immunogenic properties across all the four serotypes in the ELISA experiments, were used to produce antisera. Additionally, the whole DENV envelope obtained from DENV1 NR-4551 was used to generate antisera for the positive control where, PBS with the adjuvant was used to generate antisera for the negative control. Three Balb/c mice that are 6–8 weeks old, were immunized with each peptide/control.

Initially mice were adapted to in-house animal facility conditions prior to immunization. Initial immunization dose consisted of 50 µg of antigen with Complete Freunds adjuvant and the subsequent two boosting doses consisted of 25 µg antigen with Incomplete Freunds adjuvant. Seven days after the second boosting the mice were bled [32] to collect the antisera. The collected serum samples were stored at − 20 °C.

### Microneutralization assay for DENV

A standard Focus Reduction Neutralization test (FRNT) was modified to accommodate limited specimen availability. Briefly, 96-well cell culture plates were seeded overnight with approximately 2 × 10^4^ Vero-81 cells per well. Eight serial threefold dilutions were made of each mice antiserum sample, generated against a given peptide or the positive control, and mixed with equal volume of virus at a concentration of ~ 100 focus-forming units of virus in DMEM with 2% FBS. The mixture was incubated for 1 h at 37 °C prior to transferring 50 µl to the Vero seeded plates and incubating for 1 h at 37 °C. OptiMEM overlay media (Gibco, 31,985) supplemented with 2% FBS, 1% Anti-Anti and 5 g (1%) Carboxymethylcellulose (Sigma, C-5013) was then added and incubated for 48 h for (DENV2 and DENV4) or 51–52 h (DENV1, DENV3). Cells were fixed with 70 µl of 4% paraformaldehyde (Thermo, 28,908) for 30 min and 100 µl of permeabilization buffer was added for 10 min followed by 100 µl of blocking buffer (5% Non-Fat milk powder in permeabilization buffer). Fifty microliters of a mixture of primary antibodies (hybridoma supernatant) 4G2 and 2H2 (ATCC, HB-114) were added to the plates and incubated for a 1 h at 37 °C. Cells were washed with PBS using a cell microplate washer (BioTek, ELx405) followed by the addition of 50 µl of 1:1900 goat anti-mouse secondary antibody conjugated with horseradish peroxidase (KPL, 074-1806) for 1 h at 37 °C. Plates were washed again. Foci were stained with 60 µl of True Blue (KPL, 5510-0030) and counted. Three samples of the negative control serum were included on every plate to define the 100% infection.

### Calculation of neutralization titer 50% (NT50) for serum samples

Developed spots were read using 96 well formats of the ELISPOT machine. The mean spot value was calculated. The spot count was normalized using the negative serum sample count. Sera with > 50% neutralization at the first dilution point were further analyzed in Prism 7 (GraphPad, La Jolla California USA, www.graphpad.com). Using Graph pad prism the percentage infection was plotted against the log value of the serum dilution to calculate the NT50. NT50 values (the serum dilution effecting a 50% reduction in virus infection) were determined using the sigmoidal dose response (variable slope) equation. Sera with EC50 > 100 were considered positive for neutralizing antibodies.

## Supplementary Information

**Additional file 1: Table 1.** Peptides representing E protein epitopes with > 50% pan serotype conservancy

**Additional file 2: Table 2.** Peptides representing prM protein epitopes with > 50% pan serotype conservancy

**Additional file 3: Table 3.** Peptides representing E protein epitopes with < 50% pan serotype conservancy

## Data Availability

All data used and analyzed during the present study will be available from the corresponding author on reasonable request.

## Abbreviations

DENV: Dengue virus
E: Envelope
prM: Pre-membrane
ELISA: Enzyme Linked Immunosorbent Assay
DHF: Dengue hemorrhagic fever
C: Capsid
GLM: General linear models
ABTS: 2,2'-Azino-bis (3-ethylbenzothiazoline-6-sulfonic acid)
LS: Least square
SD: Standard deviation
DMEM: Dulbecco's modified eagle medium
FBS: Fetal bovin serum
P: Peptide
PBST: Phosphate buffered saline
IgG: Immunoglobuline G
FRNT: Focus Reduction Neutralization test
ELISPOT: Enzyme Linked Immunospot Assay
ATCC: American Type Culture Collection
ICLAS: International Council for Laboratory Animal Science
