# Supplementary material for: Natural immunogenic properties of bioinformatically predicted linear B-cell epitopes of dengue envelope and pre-membrane proteins
Source: BMC Immunol. 2021 Nov 3;22:71. doi: 10.1186/s12865-021-00462-4 (PMC8567598; doi:10.1186/s12865-021-00462-4)
Supplement: Supplementary file 1 — Additional file 1: Table 1. Peptides representing E protein epitopes with > 50% pan serotype conservancy [file 12865_2021_462_MOESM1_ESM.docx]

**Supplementary Table 1. Peptides representing E protein epitopes with > 50% pan serotype conservancy**

| **ID of the Predicted Epitope** | **# Epitope Sequence** | **Pan- serotype**  **conservancy** | **Representative peptide ID** |
| --- | --- | --- | --- |
| EP1/E | SRDFVEGLSGATW  *8-20/DI | 69 | P1/E (7-23) |
| EP2/E | SGATWV  *16-21/DI | 66 |  |
| EP3/E | CVTTMAKDKPTL  *30-41/DI | 75 | P2/E (30-46) |
| EP5/E | EAKISNTTTDSRCPTQGEATLVEEQDANFV CRRTFVDRGWGNGCGLFGKGSLITCAKFKCVT  *62-123/DII | 64 | P3/E (60-76),  P4/E (72-88),  P5/E (89-104),  P6/E (110-26) |
| EP6/E | AKISNTTTDSRCPTQGEATLVEEQDAN  *63-89/DII | 67 |  |
| EP7/E | VDRGWGNGCGLF  *97-108/DII | 83 |  |
| EP10/E | ALTLDCSPRTGLD  *180-192/DI | 54 | P7/E (178-194) |
| EP13/E | LVTFKTAHAKKQEVVVLGS  *237-255/DII | 63 | P8/E (238-254) |
| EP15/E | VLGSQEGAMHTALTGATEIQTSGTTTI  *252-278/DII & DI | 55 | P9/E (255-271) |
| EP16/E | FAGHLKCRLKMDKLTKGMS  *279-298/DI | 65 | P10/E (279-295) |
| EP18/E | FKLEKEVAETQHGT  *306-319/DIII | 50 | P11/E (308-324) |
| ¥EP19/E | KEVAETQHGTVLVQIKYEGT  *310-329/DIII | 55 |  |
| ¥EP24/E | PPFGDSYIVIGAGEKALKLSWFKKGSSIGK MF  *371-402/DIII & Stem | 59 | P12/E (371-387)  P13/E (377-393) |
| EP26/E | KKGSSIGKMFEATARGA  *393-409/ C-terminus | 71 | P14/E (394-410) |
| EP27/E | GDTAWDFGSIGGVFTSVGKL  *416-435/C-terminus | 75 | P15/E (418-434) |
| EP28/E | DTAWDFGSIGGVFT  *417-430/ C-terminus | 71 |  |
| EP29/E | IGGVFTSVGKLVHQIFGTAYG  *425-445/ C-terminus | 55 | P16/E (424-440) |
| EP31/E | IGVLLTWLGLNSRSTSLSM  *459-478/ C -terminus | 52 | P17/E (458-474) |
| EP32/E | NSRSTSL  *469-475/ C-terminus | 57 |  |

The given epitopes have been previously predicted and reported in *Nadugala et al 2016* [20], along with their pan serotype conservancy levels. Peptide sequences are based on the peptide array of strain DENV1 (Singapore/S275/1990 (NR-4551))
