# Supplementary material for: Natural immunogenic properties of bioinformatically predicted linear B-cell epitopes of dengue envelope and pre-membrane proteins
Source: BMC Immunol. 2021 Nov 3;22:71. doi: 10.1186/s12865-021-00462-4 (PMC8567598; doi:10.1186/s12865-021-00462-4)
Supplement: Supplementary file 2 — Additional file 2: Table 2. Peptides representing prM protein epitopes with > 50% pan serotype conservancy [file 12865_2021_462_MOESM2_ESM.docx]

Supplementary Table 2. Peptides representing prM protein epitopes with > 50% pan serotype conservancy

| **ID of the predicted Epitope** | **Epitope sequence** | **Pan- serotype**  **conservancy** | **Representative peptide ID** |
| --- | --- | --- | --- |
| EP1/prM | FHLTTRGGE  *1-9 | 55 | P1 (1-16)  P2 (15-31 |
| EP2/prM | TTRGGEPHMIVSKQERG  *4-20 | 59 |  |
| EP4/ prM | KTAEG  *26-30 | 60 | P2 (15-31) |
| EP6/prM | LCEDTMTYKCPRITEAEPDDVDCWCNATDTWVTYGTCSQTGEHRRDKRSV  *44-93 | 68 | P3 (43-57),  P4 (55-74),  P5 (73-90) |
| EP8/ prM | VALAPHVGLGLETRTETWMS  *93-112 | 75 | P6 (88-107) |
| EP9/prM | LETRTETWMSSEGAWKQIQKV  *103-123 | 57 | P7 (102-119) |
| EP10/prM | TWALR  *125-129 | 60 | P8 (126-142) |
| EP11/ prM | ALRHPGFTIALFLAHAIGT  *127-146 | 50 |  |

*The given epitopes have been previously predicted and reported in Nadugala et al 2016 [20], along with their pan serotype conservancy levels. Peptide sequences are based on the peptide array of the strain DENV2 (New Guinea C (NR-506))*
