# Supplementary material for: Natural immunogenic properties of bioinformatically predicted linear B-cell epitopes of dengue envelope and pre-membrane proteins
Source: BMC Immunol. 2021 Nov 3;22:71. doi: 10.1186/s12865-021-00462-4 (PMC8567598; doi:10.1186/s12865-021-00462-4)
Supplement: Supplementary file 3 — Additional file 3: Table 3. Peptides representing E protein epitopes with < 50% pan serotype conservancy [file 12865_2021_462_MOESM3_ESM.docx]

**Supplementary Table 3. Peptides representing E protein epitopes with < 50% pan serotype conservancy**

| **ID of the Predicted Epitope** | **Epitope Sequence** | | **Pan- serotype**  **Conservancy %** | | **Representing peptides (length of the peptide)** |
| --- | --- | --- | --- | --- | --- |
| EP4/E | TEVTNPAV  *48-55/DI&DII | | 13 | | DENV1:48-**T**EVTNP**A**VLRKLCIEAK-64 (17) |
|  |  |  |  |  | DENV2:45-LIE**T**EAKQP**A**TLRKYCI-61 (17) |
|  |  |  |  |  | DENV3:44-ELQK**T**EATQL**A**TLRKLCI-61(18) |
|  |  |  |  |  | DENV4:42-DFELTK**T**TAKEV**A**LLRTY-59(18) |
| EP8/E | HTGDQHQVGNESTEHGTTATITPQAPTTEIQLT  *144-176/DI | | 42 | | DENV1:144-HTGDQHQVGNETTEHGT-160(17) |
|  |  |  |  |  | DENV2:141-TPHSGEEHAVGNDTGKH-157(17) |
|  |  |  |  |  | DENV3:142-TVHTGDQHQVGNETQGV-158(17) |
|  |  |  |  |  | DENV4:142-TVHNGDTHAVGNDTSNH-158(17) |
| EP9/E | PQAPTTEIQLTDYGALTL  *165-182/DI | | 35 | | DENV1:165-**P**QAPTS**E**IQ**L**TD**YG**ALT-181(17) |
|  |  |  |  |  | DENV2:160-EIKITPQSSITEAELTGY-177(18) |
|  |  |  |  |  | DENV3:165-**P**QASTV**E**AI**L**PE**YG**TLGL-182(18) |
|  |  |  |  |  | DENV4:161-AMIT**P**RSPSVEVKLPD**Y**-177(17) |
| Ep11/E  EP12/E | LPWTSGASTSQETWNR  *218-233/DII  GASTSQETW  *223-231/DII | | 37  44 | | DENV1:220-WTSGASTSQETWNRQDL-236(17) |
|  |  |  |  |  | DENV2: 212-WFLDLPLPWLPGADTQGSNW-231(20) |
|  |  |  |  |  | DENV3:215-DLPLPWTSGATTETPTW-231(17) |
|  |  |  |  |  | DENV4:213-FLDLPLPWTAGADTSEVH-230(18) |
| EP14/E | | TAHAKKQ  *242-248/DII | | 43 | DENV1:232-NRQDLLVTFKTAHAKKQ-248(17) |
|  |  |  |  |  | DENV2:237-LVTFKNPHAKKQDVVVL-253(17) |
|  |  |  |  |  | DENV3:237-LVTFKNAHAKKQEVVVL-253(17) |
|  |  |  |  |  | DENV4:237MVTFKVPHAKRQDVTVL-253(17) |
| EP17/E | | LKMDKLTLKGMSYVMCTGSFKLEKEVA  *287-313/DI&DIII | | 48 | DENV1:285-CRLKMDKLTLKGMSYVM-301(17) |
|  |  |  |  |  | DENV2:286-RLRMDKLQLKGMSYSM-301(16) |
|  |  |  |  |  | DENV3:282-HLKCRLKMDKLELKGMSY-299(18) |
|  |  |  |  |  | DENV4:282-HLKCRLKMDKLELKGMSY-299(18) |
| EP20/E | | VQIKYEGTDAPCKIPFSTQDEKGVTQNG *322-349/DIII | | 35 | DENV1:325-KYEGTDAPCKIPFSTQD-341(17) |
|  |  |  |  |  | DENV2:322-VQIKYEGDGSPCKIPFEIM-338(19) |
|  |  |  |  |  | DENV3:326-YKGEDAPCKIPFSTEDGQGK-345(20) |
|  |  |  |  |  | DENV4:320-TVVKVKYEGAGAPCKVPI-337(18) |
| EP21/E | | YEGTDAPCKIPFSTQDEKGVTQNGRLIT  *326-353/DII | | 36 | DENV1:337-FSTQDEKGVTQNRLITA-353(17) |
|  |  |  |  |  | DENV2:338-IMDLEKRHVLGRLITV-353(16) |
|  |  |  |  |  | DENV3:335-PFSTEDGQGKAHNGRLI-351(17) |
|  |  |  |  |  | DENV4:334-VPIEIRDVNKEKVVGRLI-351(18) |
| EP22/E  EP23/E | | PIVTDKEKPVNIEAEPPFGES  *356-376/DIII  PIVTDKEKPV  *356-365/DIII | | 43  10 | DENV1:356-PIVTDKEKPVNIETE-370(15) |
|  |  |  |  |  | DENV2:352-ITVNPIVTEKDSPVNIEA-369(18) |
|  |  |  |  |  | DENV3:356-PVVTKKEEPVNIEAEPPF-373(18) |
|  |  |  |  |  | DENV4:355-TPLAENTNSVTNIEL-369(15) |
| EP25/E | | IGAGEKA  *380-386/DIII | | 29 | DENV1:371-PPFGESYIVVGAGEKAL-387(17) |
|  |  |  |  |  | DENV2:380-IGVEPGQLKLNWFKK-394(15) |
|  |  |  |  |  | DENV3379-VIGIGDKALKINWYKK-394(16) |
|  |  |  |  |  | DENV4:373-FGDSYIVIGVGNSALTLH-390(18) |
| EP30/E | | TMKIGIGVLLTWLGLNSRSTSLSMTCIAVGLITLY  *454-488/ C-terminus | | 42 | DENV1:453-WTMKIGIGILLTWLGLN-469(16)  :471-RSTSLSMTCIAVGMVTL-487(17) |
|  |  |  |  |  | DENV2:450-GVSWIMKILIGVIITWI-466(17)  :470-SRSTSLSVSLVLVGVVTL-487(18) |
|  |  |  |  |  | DENV3:450-GVSWIMKIGIGVLLTWI-469(17)  468-LNSKNTSMSFSCIVIGII-485(18) |
|  |  |  |  |  | DENV4:450-GVSWMIRILIGFLVLWI-466(17)  468-TNSRNTSMAMTCIAVGGI-485(18) |

*The given epitopes have been previously predicted and reported in Nadugala et al 2016 [20], along with their pan serotype conservancy levels. The corresponding epitope/peptide sequences from cross serotypes are based on the peptide arrays; DENV1 (Singapore/S275/1990 (NR-4551)), DENV2 (New Guinea C (NR-507)), DENV3 (Sleman/1978 (NR-511)), DENV4 (Dominica/814669/1981 (NR-512)).*
